# Functional Characterization of the Putative POT from *Clostridium perfringens*

**DOI:** 10.3390/biology12050651

**Published:** 2023-04-26

**Authors:** Hani Gharabli, Maria Rafiq, Anna Iqbal, Ruyu Yan, Nanda G. Aduri, Neha Sharma, Bala K. Prabhala, Osman Mirza

**Affiliations:** 1 Department of Drug Design and Pharmacology, Faculty of Health and Medical Sciences, University of Copenhagen, Universitetsparken 2, DK-2100 Copenhagen, Denmark; hghara@biosustain.dtu.dk (H.G.); maria.rafiq@sund.ku.dk (M.R.);; 2 Department of Physics, Chemistry and Pharmacy, Faculty of Science, University of Southern Denmark, Campusvej 55, DK-5230 Odense, Denmark

**Keywords:** major facilitator superfamily, POTs, peptide transport, *Clostridium perfringens*

## Abstract

**Simple Summary:**

Secondary active transporters have major roles in transporting vital nutrients and other smaller molecules in and out of cells. Proton-coupled oligopeptide transporters comprise a family of secondary transporters with specificity towards very short peptides and a very large number of species contain these transporters. In bacteria, proton-coupled oligopeptide transporters play different physiological roles. However, they have also been shown to import antibiotics and other drugs; hence, these transporters are of particular interest in bacteria. Here we sought to characterize such a transporter from the bacterium *Clostridium perfringens*, the only and previously uncharacterized proton-coupled oligopeptide transporter from this bacterium. Our findings were surprising as conventional proton-coupled oligopeptide transport was low, but substrate exchange, an inducible transport mode observed among similar transporters, was more prominent. This finding was investigated in light of mutational studies based on the predicted 3D structure of the transporter.

**Abstract:**

Proton-coupled oligopeptide transporters (POTs) are a fundamental part of the cellular transport machinery that provides plants, bacteria, and mammals with nutrition in the form of short peptides. However, POTs are not restricted to peptide transport; mammalian POTs have especially been in focus due to their ability to transport several peptidomimetics in the small intestine. Herein, we studied a POT from *Clostridium perfringens* (CPEPOT), which unexpectedly exhibited atypical characteristics. First, very little uptake of a fluorescently labelled peptide β-Ala-Lys-AMCA, an otherwise good substrate of several other bacterial POTs, was observed. Secondly, in the presence of a competitor peptide, enhanced uptake of β-Ala-Lys-AMCA was observed due to trans-stimulation. This effect was also observed even in the absence of a proton electrochemical gradient, suggesting that β-Ala-Lys-AMCA uptake mediated by CPEPOT is likely through the substrate-concentration-driving exchange mechanism, unlike any other functionally characterized bacterial POTs.

## 1. Introduction

Proton-coupled oligopeptide transporters (POTs) belong to the major facilitator superfamily. Several peptide transporters have been cloned, expressed, and characterized in order to understand the underlying transport mechanism of POTs [1]. In addition to di- and tripeptide uptake, they have been associated with the transport of numerous orally administered drugs such as β-lactam antibiotics, antivirals, and other peptidomimetic drugs [2]. In mammals, four POTs have been cloned and expressed, namely PepT1 (SLC15A1) [3,4], PepT2 (SLC15A2) [5,6], PHT2 (SLC15A3) [7,8], and PHT1 (SLC15A4) [7]. PepT1 is mainly expressed in the small intestine and responsible for the uptake of nutrient di- and tripeptides from dietary protein, while PepT2 is expressed in the kidney and prevents peptide loss in tubular filtrate [9]. PepT1 and PepT2 have several bacterial orthologs which provide robust systems for the characterization and structural studies of POTs. The prototypical bacterial peptide transporters, like their human counterparts, transport both di- and tripeptides [10].

Studies on bacterial POTs have yielded several crystal structures [11,12,13,14,15,16,17,18], showing that POTs exhibit the MFS fold observed in other major facilitator superfamily members. The twelve transmembrane domains (TM) form two six-helix domains, the N-terminal domain (TM1-6) and C-terminal domain (TM7-12). Bacterial POTs contain two additional helices connecting helix 6 and helix 7, forming a membrane-embedded hairpin not observed in the structures of mammalian POTs [19,20]. The N- and C-terminal domains create a large cavity opening towards the extracellular surface allowing substrates and protons to bind, inducing conformational changes for substrate transport [20]. These conformations are thought to be controlled by a periplasmic and a cytoplasmic salt bridge connection between the two domains which control and stabilize the conformational changes of POTs [21]. 

However, some POTs have been demonstrated to possess alternative characteristics compared to the ‘prototypical’ POTs. For example, a POT from *Neisseria meningitidis* (NmPOT) was found to be unable to accommodate a positively charged residue on the C-terminal position of the substrate [22]. Another example is the POT from *E. coli* (YjdL) which displayed a significantly higher preference towards dipeptides compared to tripeptides, exhibiting unusual behavior [23].

In this study, we investigated a POT from the Gram-positive, extremely versatile pathogenic bacterium *Clostridium perfringens* (CPEPOT), which can cause a wide variety of infections in humans and livestock, including enterocolitis, enterotoxemia, and gas gangrene [24,25]. We sought to investigate its characteristics using functional and mutational studies, sequence analysis, and structural modelling. Our studies revealed that CPEPOT has functional and structural differences from prototypical bacterial POTs.

## 2. Materials and Methods

### 2.1. Protein Expression

All mutated variants of CPEPOT were synthesized and subcloned commercially (GenScript, Piscataway, NJ, USA). All transporter variants have C-terminal hexa-histidine-tag *His_6_*. All variants were overexpressed by picking a single colony from a plate of BL21 (DE3) pLysS cells transfected with a pTTQ18-plasmid-carrying transporter cDNA insertion or empty pTTQ18 vector and inoculated in 5 mL LB medium containing 34 µg/mL chloramphenicol and 100 µg/mL ampicillin. The culture was grown overnight in a static incubator at 37 °C. The following day, 10 mL LB medium, with the same antibiotic concentration, was added to the 5 mL pre-cultures and allowed to grow at 37 °C in a shaking incubator at 200–250 RPM until the cultures reached an OD_600_ of 0.6–0.8. Protein expression was induced by the subsequent addition of 1 mM IPTG. Cells were harvested 3 h post induction via centrifugation. All other described POTs including YdgR and YjdL were transformed, over-expressed, and harvested using the same procedure.

### 2.2. Western Blot

Western blotting was performed as described previously [23,26,27]. Briefly, a cell pellet was resuspended in lysis buffer (50 mM Tris-HCl, 150 mM NaCl, 1% Triton X-100, and cOmplete™ Protease Inhibitor Cocktail tablet (Roche, Basel, Switzerland) per 25 mL buffer). Samples were incubated for 30 min on ice followed by 30 s sonication (Bandelin Sonopuls mini20 homogenizer). The samples were then centrifuged for 15 min (12,000× *g* at 4 °C). The cleared lysates were separated and analyzed by SDS-PAGE (NuPage^®^ 10% bis-tris gel) with subsequent blotting onto a PVDF-membrane. The blotted membrane was blocked using blocking buffer (140 mM NaCl, 2.7 mM KCl, 10 mM Na_2_HPO_4_, 1.8 mM KH_2_PO_4_, 3% bovine serum albumin (BSA), 0.5% Tween 20) overnight at 4 °C. Immunodetection was conducted using mouse anti-His_6_ and HRP-conjugated rabbit anti-mouse antibodies (IBA) (1:2500 dilution). Band quantification was performed using ImageJ [28].

### 2.3. Uptake Assay

The harvested cell pellets were washed thrice in modified Krebs buffer (50 mM MES, 140 mM NaCl, 5.4 mM KCl, 1.8 mM CaCl_2_, 0.8 mM MgSO_4_, and 5 mM glucose, pH 6.5). The cell pellet was resuspended in a modified Krebs buffer to OD_600_ = 10. All assays employed a total sample volume of 100 µL in which 50 µL consisted of the fluorescent probe and analytes (peptides) and 50 µL consisted of the cell suspension unless stated otherwise. β-Ala-Lys-N-7-amino-4-methylcoumarin-3-acetic acid (β-Ala-Lys-AMCA) was used at a final concentration of 0.5 mM and peptides were used at 10 mM final concentration unless stated otherwise. Assays were performed on a heating block at 37 °C, 300 RPM for 10 min. Assay reactions were terminated by adding 500 µL ice-cold modified Krebs buffer followed by centrifugation (16,000× *g*, 1 min). Cell pellets were washed three time with ice-cold modified Krebs buffer followed by resuspension in 100 µL modified Krebs buffer. The samples were quantified by fluorescence measurements (excitation at 340 nm and emission at 460 nm) using a Safire^2^ plate reader (Tecan, Männedorf, Switzerland). Empty-vector (pTTQ18)-transformed *E. coli* cells were used as negative control. Data analysis was performed using GraphPad Prism version 9.1.0 for macOS (GraphPad Software, San Diego, CA, USA). All results are representative of at least three biological replicates.

### 2.4. Peptide Specificity and pH Dependence Assay

An uptake inhibition assay was performed with the addition of alanine-, di-, tri-, and tetra-alanine peptides in the buffer to compete with the β-Ala-Lys-AMCA. The peptide specificity assay was performed accordingly, with A-K, K-A, A-D, D-A, A-F, and F-A peptides. All compounds were purchased from Sigma Aldrich. The pH sensitivity assay was performed at three different pH values 5.5, 6.5, and 7.5. For reactions at pH 5.5 and 6.5, modified Krebs buffer with MES as a buffering agent was used, while at pH 7.5, HEPES was used as a buffering agent. Assays were performed with β-Ala-Lys-AMCA in the presence and absence of A-A. The total assay time was 15 min for all replicates.

### 2.5. Time-Dependent Uptake Assay

Resuspended cells were transferred to Eppendorf tubes containing 0.5 mM β-Ala-Lys-AMCA in the presence and absence of 10 mM A-A and incubated at 37 °C for 10 min at 250 RPM. An amount of 100 µL was taken from the samples at specific time points and added to an Eppendorf tube containing 500 µL ice-cold modified Krebs buffer. The experiment was also performed with the addition of CCCP (10 µM) in the presence of 10 mM A-A at time = 15 min.

### 2.6. Uptake Assays in the Absence of a Proton Electrochemical Gradient

Harvested cell pellets were resuspended in a modified Krebs buffer (OD_600_ = 10). After leaving the cells in the buffer for 20 min at RT, A-A was added to the cell suspension to a final concentration of 50 mM and incubated at 37 °C, 250 RPM for 30 min. Cells were then pelleted, washed twice, resuspended to OD_600_ = 10, and then immediately transferred to Eppendorf tubes containing 0.5 mM AMCA and in the presence and absence of 10 µM carbonyl cyanide *m*-chlorophenyl hydrazone (CCCP). The assay was terminated after 5 min by the addition of ice-cold modified Krebs buffer.

### 2.7. Bioinformatic Analysis

CPE0544 (UniProt entry Q8XMZ4_CLOPE) was used as a query in BLAST (protein BLAST, NCBI) for randomly selected prokaryotes, *Bacillus subtilis*, *E. coli*, *Shewanella oneidensis*, *Neisseria meningitidis*, *Yersinia pestis*, *Geobacillus kaustophilus*, *Streptococcus thermophilus*, *Bacillus cereus*, and *Staphylococcus hominis*. The BLAST results for each prokaryote were aligned using MAFFT [29] and filtered using Jalview [30] (filtered for unusually short or long sequences and sequences that did not contain the ExxER motif). The sequences were realigned with MAFFT after filtering. The alignments were viewed in Jalview and the sequences were divided into sequences containing a salt bridge equivalent to the YdgR periplasmic salt bridge (E56 and R305) and a hairpin, sequences containing a YdgR salt bridge equivalent but not the hairpin, sequences lacking a YdgR salt bridge equivalent but containing a hairpin, and sequences lacking both a YdgR salt bridge equivalent and a hairpin. The representation of each of the four variants was related to the total number of sequences analyzed (4796 sequences after filtration) to yield the representation as a percentage.

## 3. Results

### 3.1. Increased β-Ala-Lys-AMCA Uptake in the Presence of Di- and Tripeptides

**Figure 1:**
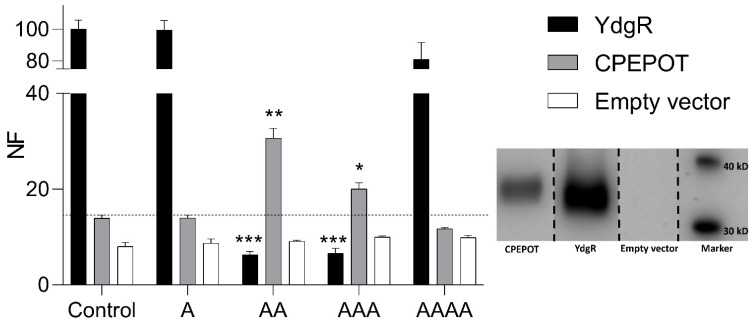
YdgR- and CPEPOT-mediated β-Ala-Lys-AMCA uptake inhibition in the presence of A, AA, AAA, AAAA, and compared to background uptake by empty vector (pTTQ18). Fluorescence normalized to the uptake of CPEPOT. Insert is a Western blot showing the expression level of each transporter protein. The blot is from the same gel but assembled for illustrational purposes. Lane 1, 2, 3, 4: CPEPOT, YdgR, empty vector, marker. NF = normalized fluorescence intensity. YdgR shows a significant decrease while CPEPOT shows a significant increase in β-Ala-Lys-AMCA uptake inhibition in the presence of AA and AAA, respectively. Significance level indicated by * for *p* < 0.05, ** for *p* < 0.02, and *** for *p* < 0.01. All experiments performed in triplicate (*n* = 3).

CPEPOT was overexpressed in *E. coli* and subjected to an uptake assay. The prototypical transporter YdgR was subjected to the same assay for comparative analysis. The YdgR-mediated uptake of β-Ala-Lys-AMCA was not outcompeted by A and AAAA, but its uptake was significantly decreased in the presence of AA and AAA which is in agreement with previous studies (Figure 1) [26,31]. CPEPOT was tested for its ability to recognize and translocate β-Ala-Lys-AMCA and this was compared with *E. coli* cells expressing an empty vector (pTTQ18) (Figure 1). Furthermore, CPEPOT was subjected to the same uptake assay as was performed with YdgR. Here, CPEPOT showed atypical functional characteristics, i.e., AA and AAA significantly increased the uptake of β-Ala-Lys-AMCA as compared to the empty vector, whereas A and AAAA did not have any effect on the β-Ala-Lys-AMCA uptake (Figure 1). Overall β-Ala-Lys-AMCA uptake by CPEPOT was significantly lower than YdgR, which could be attributed to its lower expression level as observed with the Western blot analysis (Figure 1).

### 3.2. Increased β-Ala-Lys-AMCA Uptake in the Presence of Dipeptides

**Figure 2:**
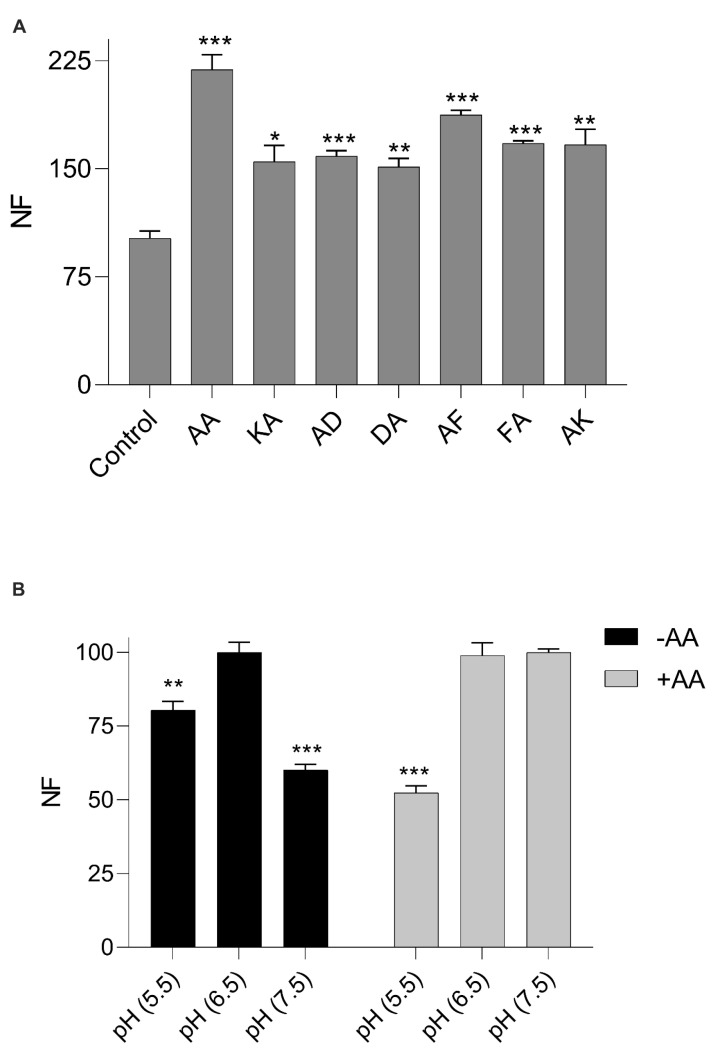
(**A**) CPEPOT-mediated uptake of β-Ala-Lys-AMCA in the presence of AA, KA, AD, DA, AF, FA, and AK. Fluorescence normalized according to control. (**B**) CPEPOT-mediated uptake of β-Ala-Lys-AMCA at pH 5.5, 6.5, and 7.5 in the absence and presence of AA. Fluorescence normalized according to the intensity at pH 6.5 in each experiment. Significance level indicated by * for *p* < 0.05, ** for *p* < 0.02, and *** for *p* < 0.01. All experiments performed in triplicate (*n* = 3).

Based on the above results, we further explored the characteristics of CPEPOT which deviated from that of the prototypical POT. A small dipeptide library (A-X or X-A) having either an acidic, basic, or aromatic side chain on the N- or C-termini with an A residue on the opposite terminal was screened. As illustrated in Figure 2A, all dipeptides enhanced the uptake of β-Ala-Lys-AMCA compared to the control where no stimulating substrate was added. Furthermore, CPEPOT did not have any preference for either charge or hydrophobicity on either of the termini.

### 3.3. pH Dependency of β-Ala-Lys-AMCA Uptake

The pH dependency of CPEPOT was investigated at three different pH values, i.e., pH 5.5, 6.5, and 7.5 in the presence and absence of AA as a stimulator. In the absence of A-A (Figure 2B), a pH of 6.5 was found to be the optimum pH as observed in previously reported POTs [32], while a significant decrease in β-Ala-Lys-AMCA uptake was observed at pH 5.5 and 7.5. In the presence of A-A, this trend was not followed since no significant change in β-Ala-Lys-AMCA uptake at pH 7.5 compared to pH 6.5 was observed, while the uptake decreased at pH 5.5 (Figure 2B).

### 3.4. Addition of a “Stimulator” Peptide Results in a Higher Activity of CPEPOT

**Figure 3:**
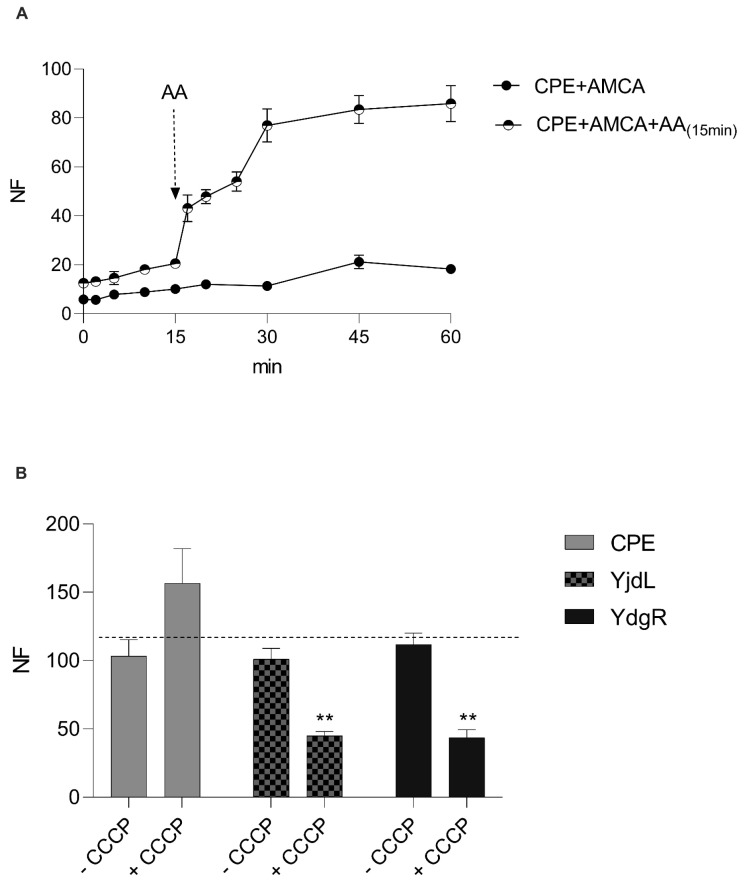
(**A**) Time-dependent CPEPOT-mediated uptake of β-Ala-Lys-AMCA with and without subsequent addition of AA at time = 15 min. (**B**) Uptake of β-Ala-Lys-AMCA in the absence and presence of CCCP (abolishing proton electrochemical gradient) by CPEPOT, YjdL, and YdgR. Fluorescence normalized according to −CCCP in each experiment. Significance level indicated by ** for *p* < 0.02. All experiments performed in triplicate (*n* = 3).

Following these results, the uptake was followed as a function of time under different conditions to attain a better understanding of CPEPOT. The uptake of CPEPOT was followed as a function of time (Figure 3A). There was minimal uptake in the absence of A-A, but addition of A-A at time = 15 min resulted in accumulation of β-Ala-Lys-AMCA until and after 60 min. The stimulatory effect happened instantly after the addition of A-A into the reaction mixture.

### 3.5. Removing the Proton Gradient Does Not Alter the Function of CPEPOT

To further investigate the reason this stimulatory effect was observed for CPEPOT, we investigated the role of the proton electrochemical gradient by adding a protonophore (CCCP). Cells were preloaded with A-A and the proton electrochemical gradient was eradicated by the addition of CCCP. The trans-stimulatory effect/exchange mechanism in CPEPOT was observed in the presence of CCCP (Figure 3B), whereas the prototypical transporters YjdL and YdgR exhibited a significant decline in uptake activity in the presence of CCCP.

### 3.6. CPEPOT Lacks the Extra Helices and Gating Salt Bridges Observed in Prototypical POTs

**Figure 4:**
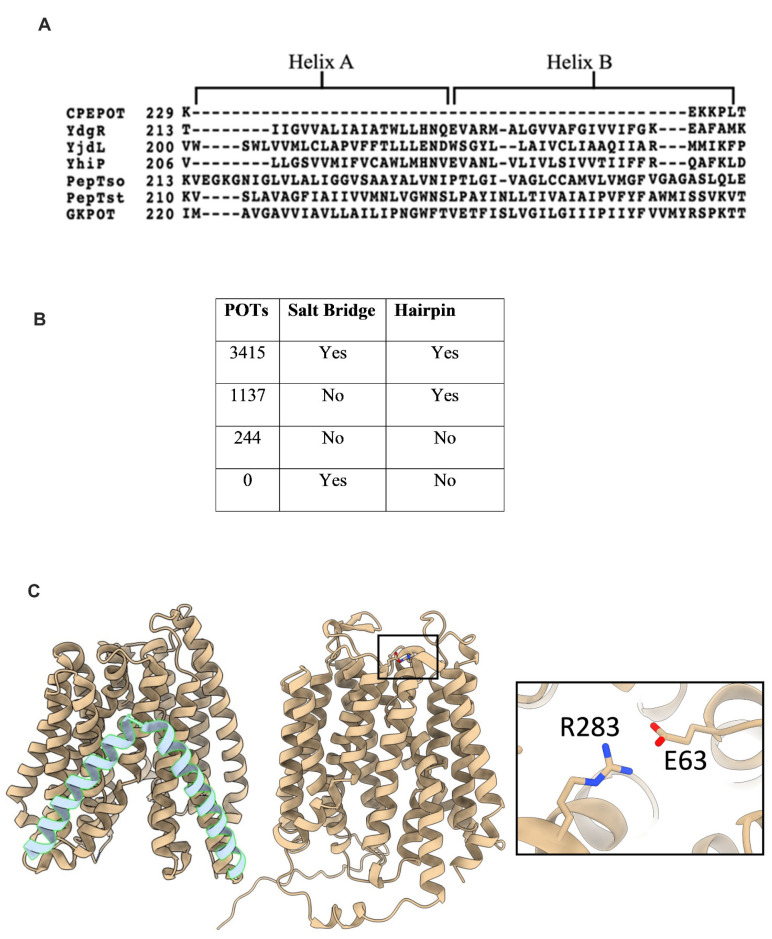
(**A**) Multiple sequence alignment of CPEPOT, YdgR, YjdL, YhiP, PepTso, PepTst, and GKPOT. (**B**) Schematic representation of the four different types of POTs: with hairpin and periplasmic salt bridge, without salt bridge but containing hairpin, with neither salt bridge nor hairpin, and without hairpin but containing salt bridge (**C**) Left: YdgR (PDB:6GS4), a canonical bacterial POT structure showing two additional helices. Middle: AlphaFold model of CPEPOT with missing additional helices. Right: Enlarged view showing induced periplasmic mutations forming salt bridge in CPEPOT.

To understand the functional results in a 3D-structural context, the AlphaFold model of CPEPOT was used [33]. Furthermore, the primary sequence of CPEPOT was aligned with sequences of known bacterial POTs to find discrepancies in their primary structure. The alignment revealed a large section missing in CPEPOT (Figure 4A) which encodes the two additional alpha-helices between N- and C-terminal domains in other bacterial POTs between transmembrane helices 6 and 7. These results are well in agreement with the AlphaFold homology model of CPEPOT, which also confirmed the absence of the two additional alpha-helices in CPEPOT (Figure 4C). Another noteworthy difference is that POTs contain gating salt bridges that stabilize the outwards and inwards conformations [21], while in CPEPOT none of these salt bridges appear to be conserved. This analysis was extended to alignments of organism-specific POTs to investigate the conservation of the salt bridge residues across prokaryotes. Further, we investigated a connection between having a hairpin and having a periplasmic salt bridge, which is conserved in YdgR. These results show that the most predominant POT species, 71% (3415), contain both the hairpin and periplasmic salt bridge (Figure 4B), but the presence of the hairpin does not guarantee the presence of a salt bridge as 24% (1137) were found to only have the hairpin conserved (Figure 4B) whereas 5% (244) of the POTs do not have salt bridges and hairpins. CPEPOT falls within this 5% as it has neither a salt bridge nor a hairpin. To our knowledge, this is the first functional study of such a bacterial POT.

### 3.7. Addition of a Periplasmic Salt Bridge Does Not Alter the Function of CPEPOT

**Figure 5:**
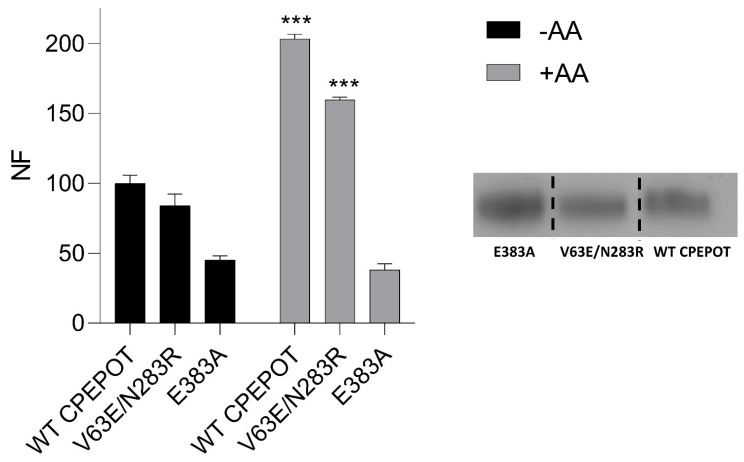
Uptake inhibition assay of mutants V63E/N283R and E383A in the presence and absence of trans-stimulator AA. Fluorescence normalized to CPEPOT in the absence of AA. Western blot of mutant transporter proteins E383A, V63E/N283R, and WT CPEPOT as an insert. Each band is representative of three independent biological replicates *n* = 3. The blot is from the same gel but assembled for illustrational purposes. In the presence of AA, WT CPEPOT and V63E/N283R show a significant increase in the uptake of β-Ala-Lys-AMCA. Significance level indicated by *** for *p* < 0.01. All experiments performed in triplicate (*n* = 3).

To further explore the role of periplasmic salt bridge gating in the functional activity of CPEPOT, equivalent to YdgR periplasmic salt bridge (E56 and R305) [34], double mutations V63E and N283R were introduced in CPEPOT. The mutations were observed to be facing each other in the CPEPOT AlphaFold model (Figure 4C). Furthermore, we also studied a mutant missing a previously described vital binding site residue, E383A, to kill the functionality of CPEPOT [26,35]. An uptake inhibition assay was performed for both V63E/N283R and E383A. Comparing V63E/N283R to WT, the stimulatory effect of A-A was conserved, though with a slight decrease in the activity, whereas E383A portrayed an activity equivalent to the background activity of the pTTQ18 empty vector, which indicated an abolished activity (Figure 5).

## 4. Discussion

Here we report a POT from *C. perfringens* which displays trans-stimulation by di- and tripeptides to facilitate uptake of β-Ala-Lys-AMCA, a well-known substrate of prototypical transporters [17], in an exchange-like manner. 

Although a *trans*-stimulatory effect was observed, the results still suggest that CPEPOT follows the same promiscuity known in the POT family since the stimulatory effect was conserved when assaying with dipeptides containing sidechains with distinct physicochemical properties and sizes (Figure 2A). Previously, an exchange mechanism in the peptide transporters was already reported in hPEPT1 by [36]. The exchange mechanism is a proton-independent process, which means that the transporter can catalyze the exchange of two substrates without net movements of protons. Exchange is known to be faster than normal proton-coupled symport, which is why the reaction was terminated after 5 min [37,38]. Our results show there is an obvious difference in pH dependency when comparing β-Ala-Lys-AMCA uptake in the absence and presence of AA (Figure 2B). Seeing that the pH optimum at 6.5 observed in the absence of AA is diminished in the presence of AA indicates that β-Ala-Lys-AMCA uptake in the presence of AA is independent of the proton gradient. It has been seen previously that for some proton-coupled symporters, the substrate drives the protonation of the transporter [39]; the same thing could be happening in the case of CPEPOT. In the presence of CCCP, we observed an improved uptake of β-Ala-Lys-AMCA, while the trend was opposite in YdgR and YjdL. This could again be an argument towards the idea of CPEPOT being more naturally adapted to exchanging substrates compared to proton-coupled transport. In the absence of CCCP, the transporter could rely on both proton-coupled transport and exchange, but the presence of CCCP ruled out slow proton-coupled transport. To establish whether any of the activities observed were related to the proposed active site of POTs, we mutated a conserved and well-studied glutamate (E383A) [26] and found it to be inactive (Figure 5).

To investigate CPEPOT’s preference for exchange, we took a structural approach. By aligning the sequence of CPEPOT with other POTs across the phylogeny, it was clear that CPEPOT was different from most POTs; CPEPOT lacks the inter domain hairpin and stabilizing salt bridges on both sides of the membrane (Figure 4C). Among these salt bridges, the periplasmic appears to be the most conserved; hence, we introduced this V63E/N283R salt bridge into CPEPOT. However, no change in activity was observed. This suggests that CPEPOT could be functioning independently of salt bridges.

## 5. Conclusions

The present study suggests that CPEPOT is an atypical POT with an unusual preference towards exchange. The structure–function relationship was investigated with mutation analysis, which led to the functional analysis of a non-naturally occurring POT with a periplasmic salt bridge. The mutant with the salt bridge behaved similar to the WT, while a mutation in the binding site abolished the activity.

## Data Availability

Not applicable.
